# Is There Still a Place for Reconstructive Surgery in Distal Tubal Disease?

**DOI:** 10.3390/jcm11123278

**Published:** 2022-06-08

**Authors:** Bogdan Obrzut, Marzanna Obrzut

**Affiliations:** 1 Department of Obstetrics and Gynecology, Institute of Medical Sciences, Medical College, University of Rzeszów, Rejtana 16 C, 35-959 Rzeszow, Poland; 2 Center for Diagnostic Medical Sonography, Litewska 4/4, 35-302 Rzeszow, Poland; marzannaobrzut@gmail.com

**Keywords:** tubal disease, peritubal adhesions, hydrosalpinx, tubal infertility, reproductive surgery

## Abstract

Tubal diseases account for 25–40% of female factor infertility. Mainly, they involve the distal part of the fallopian tube, and hydrosalpinx is the most severe manifestation. Usually, the management decision is made between reconstructive surgery and ART, depending on the severity of the tubal damage, patient age, ovarian reserve, and seminogram, as well as financial, religious, ethical, and psychological factors. Estimated live-birth rates after corrective surgery range from 9% to 69%. The success rate of IVF is about 30% live-birth rate per cycle initiated in women across all ages with tubal factor infertility. Surgery offers a long-term cure and patients may attempt conception many times but are burdened with perioperative adverse events. IVF bypasses potential complications of operative treatment; however, this has its own unique risks. The effectiveness of reconstructive surgery versus ART has not been adequately evaluated. The success of fertility management depends on a thorough interpretation of existing data and careful patient selection. The presented review provides updates on the most recent progress in this area.

## 1. Introduction

Tubal factor infertility is one of the most frequent causes of female infertility. Despite the rising usage of artificial reproductive technologies, surgery remains an important therapy option among this group of patients. However, the effectiveness of tubal reconstructive surgery against another treatment approaches has not been appropriately evaluated. There are no randomized controlled trials that compare surgery versus IVF or expectant management. Clinical practice is guided on the basis of observational studies. Researchers use different classifications and inclusion criteria. Surgical techniques are not uniform. As a result, published data differ substantially and interpreting of outcomes is consequently made more difficult. The aim of this review is to offer a comprehensive update on current evidence and guidance as well as future challenges.

## 2. Etiopathogenesis and Morphology of Distal Tubal Disease

Tubal factor infertility is responsible for 25–40% of female infertility [1,2,3,4]. Damage can involve the proximal, distal, or entire tube [5]. Most frequently, tubal disease occurs in the distal segment (about 80%) manifesting as hydrosalpinx, while in 10–25% it affects the proximal section of the fallopian tube [6]. Gebeh and Metvally divide fallopian tube disease with subsequent obstruction into three groups: proximal, mid-segment, and distal segment blockage [1]. Proximal tubal obstruction can be caused by amorphous debris and mucus plugs, pelvic inflammatory disease, salpingitis isthmica nodosa, endometriosis, obliterative intraluminal fibrosis, uterine synechiae, fibroids, or polyps situated over the tubal ostium. Mid-segment tubal blockage is usually connected to previous surgery, tubal sterilization, partial salpingectomy for ectopic pregnancy, or may be a congenital segmental absence. Reasons for distal segment obstruction include pelvic inflammatory disease, endometriosis, and post-surgical adhesions [1].

The most common cause of tubal damage is pelvic inflammatory disease, responsible for more than 50% of cases [3]. PID usually results from prior sexually transmitted disease due to *Chlamydia trachomatis* or *Neisseria gonorrhoeae* [7]. Polymicrobial infection occurs in 30–40% of cases [8]. Inflammation leads to the destruction of ciliated cells of the tubal endothelium, especially in the ampullary and infundibular sections. These specialized cells are crucial for the transport of both gametes and embryo, and often are unable to recover even after resolution of the infection. Loss of ciliated endothelial cells and post-inflammatory fibrosis of the wall impair the physiologic function of the salpinges while intraluminal and peritubal adhesions can cause occlusion of the fimbrial end. Unable to drain, the fallopian tube accumulates fluid and distends. Despite clinically successful treatment of the infection, the risk of persistent tubal damage varies between 8 and 12%. A second episode of PID increases this risk twofold, and a third episode up to 54% [1].

Endometrial lesions involve the salpinges in 6% of women with endometriosis and endometriosis-related adhesions affect the fallopian tubes in up to 26% of cases [9]. Based on the location of implants, tubal endometriosis is divided into serosal/subserosal and intraluminal [10]. In the first case, endometriotic implants are seen on the peritoneal surface of the salpinges. Cyclic local hemorrhages in the implants cause fibrosis and scarring of the tubes. In the less common intraluminal endometriosis, ectopic lesions occur on the mucosal surface of the tube wall. Repeated hemorrhages of the implants can lead to distention of the salpinx.

Regardless of the reason, hydrosalpinx is usually asymptomatic; however, some patients may present with lower abdomen pain [8]. Most frequently, it is detected accidentally or during work-up for infertility. In 10–13%, hydrosalpinges are diagnosed during ultrasound examination [11,12]. Equally, up to 30% of cases are discovered during hysterosalpingography (seen as dilated contrast-filled tube, with absence of free spillage), laparoscopy, and laparotomy [13,14,15]. Incidentally, hydrosalpinx can also be seen in a CT scan, as a fluid-attenuation tubular juxtauterine structure, separate from the ovaries [8].

The typical US image of hydrosalpinx is a complex, C- or S-shaped anechoic tubular structure, with a thin or thick wall [8]. It reveals incomplete septa that result from the distended tube folding. The pathognomonic features for hydrosalpinx are thickened longitudinal folds producing a “cogwheel” appearance [16]. Hydrosalpinx is usually well separated and distinct from both ovary and uterus [8].

In cases in which adnexal mass cannot be sufficiently evaluated with US, MR imaging remains the method of choice. On MR images, a dilated fallopian tube is seen as a fluid signal intensity tubular structure (i.e., hypointensity on T1-weighted and hyperintensity on T1-weighted images) with incomplete septa [8].

## 3. Treatment Strategy and Decision-Making Process

Decisions on the treatment of distal tubal disease are complex and difficult, and as such require a patient-specific approach. During the decision-making process, all known fertility-related factors should be analyzed. They include not only the severity of the tubal damage but also patients’ age, ovarian reserve, seminogram, and previous/concomitant disorders. Discussing patients’ safety, prior abdominopelvic operations, risk of surgical complications, and ectopic pregnancy should be taken into consideration. Surgeon experience and the estimated success rate of all treatment options (operative tubal repair, expectant management, and ART) are equally important. Patients’ factors including predicted treatment costs and insurance reimbursement, religious beliefs, and individual preferences may also have a substantial impact on the final decision [2,6]. Central, however, to patient satisfaction and avoiding potential conflict in case of unsuccessful treatment is open communication and active patient involvement during the entire process of decision making and management.

The decision whether to repair or to remove salpinges with distal disease is usually made intraoperatively based on assessment of tubal damage severity. To assist surgeons, several clinical classification systems have been developed [17,18,19,20].

One of the first that found broader application was the American Fertility Society classification of distal tubal occlusion [21]. The AFS classification involves such parameters as: distal ampullary diameter, tubal wall thickness, mucosal folds at the neostomy site, extent of adhesions, and type of adhesions. All those parameters are assigned values. According to this scoring system, tubal damage is qualified as mild (1–8 points), moderate (9–10 points), or severe (>10 points) [21,22]

The next scale to gain general acceptance among clinicians was the staging of tubal disease of Winston and Margara (WM) separating fertile women into four categories (Table 1) [23]. The WM staging system was a modified version of the Boer–Meisel classification [24].

Based on their own experiences, the authors stated that the outcome of tubal repair is determined by degree of mucosal damage, tubal fibrosis, as well as the quality of tubal and ovarian adhesions. The proposed system was effective for pregnancy prognosis.

Probably the most popular staging system from the practical point of view is the classification of tubal damage proposed by Hull and Rutherford (HR) in 2002 (Table 2) [25].

**Figure 1:**
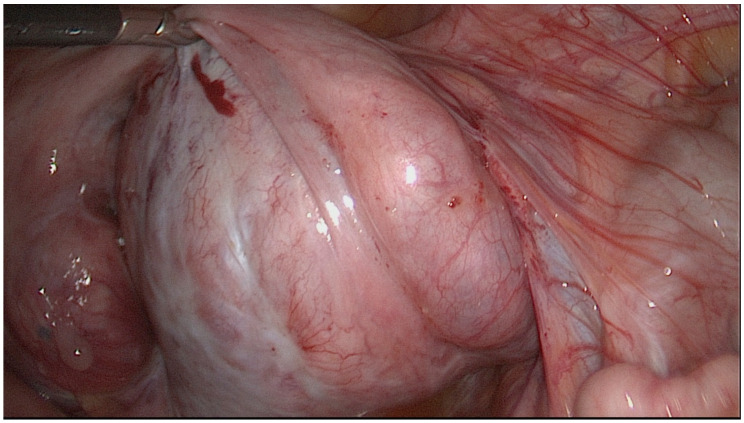
Minor tubal disease according to classification by Hull and Rutherford.

**Figure 2:**
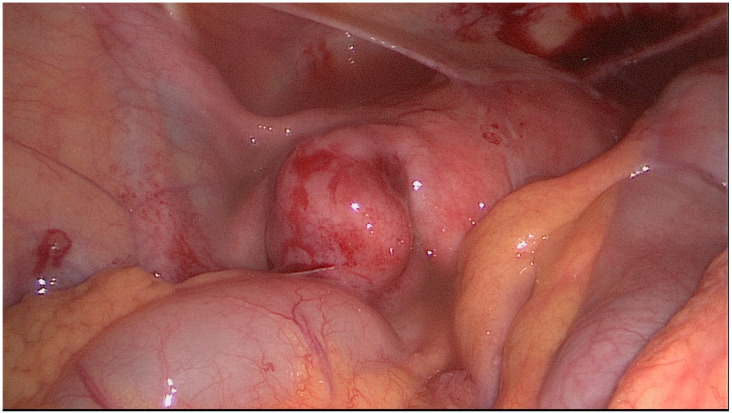
Intermediate tubal disease according to classification by Hull and Rutherford.

**Figure 3:**
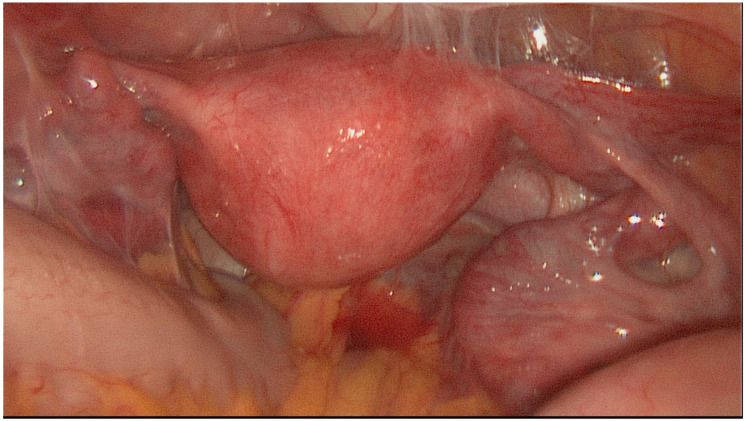
Severe tubal disease according to classification by Hull and Rutherford.

The HR classification is a relatively simple classification system reduced to three severity categories. Figure 1, Figure 2 and Figure 3 show exemplary intraoperative photographs of these three stages of tubal damage. Similarly to the WM staging system, the HR classification defines severity grades of the tubal disease in a descriptive way without using a point system. According to the findings of Akande et al., the HR classification is a reliable tool for prediction of the outcome of tubal surgery based on the severity of tubal disease [26]. Both the WM staging system and HR classification are criticized for subjectivism in assessment of the stages of tubal disease.

In 2014, Zou et al. published another classification for prognosis in tubal factor infertility [27]; this is a combination of the American Society for Reproductive Medicine’s revised classification of endometriosis, Hulka system and the Hull and Rutherford classification [17,25,28]. This is a scoring system that involves the following parameters: adhesion range, nature of adhesions, tubal patency, morphology of a tube, and fimbrial structure. All those parameters are assigned point values from 0 to 8. According to the proposed scale, tubal factor infertility (TFI) is defined as mild (0–7 points), moderate (8–15 points), or severe (>16 points). Zou et al. confirmed the effectiveness of their own classification for pregnancy prognosis based on a retrospective study including 1290 patients [27].

## 4. Operative Techniques in Reconstructive Distal Tubal Surgery

The principal goal of surgical treatment is to restore the normal anatomy of the tubes and their functional integrity. The main surgical procedures include adhesiolysis (salpingo-ovariolysis), fimbrioplasty, and neosalpingotomy.

Periadnexal adhesions interfere with the anatomic relationship between the fimbrial end of the tube and surface of the ovary and impair the act of oocyte capture. Adhesiolysis aims at the operative removal of scar tissue from around both the ovary and the salpinx and restoration of the normal anatomy.

Fimbrioplasty is applied in case of fimbrial stenosis. Its goal is to open or widen the distal end of the tube. It may involve deglutination of the fringes, dilatation of the external ostium and/or adhesiolysis for fimbrial adhesions. If necessary, the fimbrial end should be everted and ligated to the distal tubal serosa to minimize the risk of reocclusion.

Neosalpingotomy is the most advanced procedure of tubal reconstructive surgery and means the creation of a new tubal opening. First, the ampullary portion of the fallopian tube is distended by intrauterine administration of the contrast medium and the occluded ostium is identified. Then, the fallopian tube is opened in the avascular area by three to four incisions with scissors, or alternatively by electrosurgery or laser [6,29]. After a new opening is formed, the edges of the distal tube are everted and sutured using 3.0–6.0 suture to the proximal serosa of the salpinx circumferentially. Preferred are nonabsorbable monofilament sutures, as they may be less likely to elicit an inflammatory response with subsequent secondary adhesions. Eversion of the edges may also be achieved by superficial coagulation of the serosal surface of the fallopian tube using bipolar or laser energy; however, this method seems to be less effective and connected with a higher risk of reocclusion [6,30,31].

Initially, the reconstructive tubal surgery was carried out microsurgically by laparotomy [23,32,33,34]. Currently, it is reckoned that both fimbrioplasty and neosalpingostomy should be performed via laparoscopy because of comparable efficacy and lower risk of adverse events [35,36,37]. A meta-analysis of five nonrandomized controlled trials revealed a pooled intrauterine pregnancy rate of 28.9% in patients who underwent laparoscopic operation and 30.9% in women after open procedure. The difference was not statistically significant. The intrauterine pregnancy rates in mild hydrosalpinx subgroup after laparoscopic and laparotomic repair were 39.5% and 32.8%, respectively. Additionally, those results did not differ significantly [38].

Regardless of the operative technique, the crucial element of each reproductive surgery is the prevention of secondary adhesions. Numerous studies demonstrated reduced de novo adhesion formation after laparoscopic procedures compared to laparotomy [39,40,41,42,43,44,45,46]. Traditionally, this was explained by avoiding tissue desiccation as a cause of inflammatory reaction with subsequent adhesion formation and minimalization of mechanical serosal damage, which is a prerequisite for adhesion development [1]. Multiple reports from recent years have shown that oxidative stress, metabolic state, hypoxia, as well as genetic factors may play an important role in the postoperative adhesion formation [47,48]. The current understanding of the pathogenesis of pelvic adhesions is reflected in devising agents for postoperative adhesion prevention. Approval of the U.S. FDA for the reduction in postoperative adhesions received oxidized regenerated cellulose, 4% icodextrin solution, modified hyaluronic acid, and carboxymethylcellulose [49]. The application of an adhesion barrier should be considered, especially for patients with endometriosis or pelvic inflammatory disease as being at high risk of forming clinically significant adhesions [50]. Another preventive strategy is the separation of the structures during the 3–5-day healing process, considered as critical for adhesion development [49]. In reproductive surgery, this means temporary ovarian suspension to keep them separate from the pelvic side wall peritoneum or other pelvic organs. Although some reports demonstrate the effectiveness of this procedure in reduction in the rate and severity of postoperative adhesions, this still requires further investigations [49,51,52,53].

## 5. Reproductive Outcomes

Pregnancy rates after reconstructive distal tubal surgery strictly depend on the severity of tubal disease. Patients with periadnexal adhesions and patent tubes have the most favorable prognosis. Numerous studies indicate that about 80% of women with periadnexal adhesions have normal endosalpinx. Within 1 year after laparoscopic adhesiolysis, about 70% of these women will be pregnant and have a term delivery [54,55,56,57]. According to another report, spontaneous intrauterine pregnancy within 2 years after adhesiolysis for mild adhesions is 72.09%, while in the case of moderate and severe adhesions it is 51.95% and 27.91%, respectively [58].

Fimbrioplasty, another reconstructive technique, offers high success rates. In a large case series of 273 patients, Tran reports 79.8% pregnancy rate and 71.5% live-birth rate after this procedure [59].

Reproductive outcomes after neosalpingostomy markedly differ depending on the extent of tubal damage. Meta-analysis of 22 observational studies from 1972 to 2014, including 2810 patients who underwent salpingoneostomy for hydrosalpinx, revealed a pooled natural clinical pregnancy rate of 27% with a pooled live-birth rate of 25% [60]. Surprisingly, the clinical pregnancy rate in women with bilateral salpingoneostomy was 29%. It is worth emphasizing that the cited meta-analysis did not investigate the correlation between the pregnancy rate and severity of tubal disease which is essential for an objective interpretation of the results. Reproductive outcomes after neosalpingectomy are much more favorable in good-prognosis cases. As good prognosis is considered a patient with limited filmy periadnexal adhesions, only mildly dilated salpinges (<3 cm) with thin pliable wall, and a lush, normally folded mucosa [21]. Intrauterine pregnancy rates after salpingostomy for mild hydrosalpinx range from 58% to 77% [61]. Winston et al. reported a live-birth rate of 39% in women after salpingostomy for tubal disease stage I, and only 9% in stage III [23]. In a retrospective study including 3254 patients, an intrauterine pregnancy rate of 72.8% and live-birth rate of 66.8% were reported for neosalpingostomy and salpingo-ovariolysis [62]. In other research evaluating 434 women, clinical pregnancy rate was lower, showing a strong correlation with the severity of tubal disease: 43% in stage I, 33.6% in stage II, 19.5% in stage 3, and 13.8% in stage 4 [31]. Zhou et al., in a study including 1290 patients treated operatively for tubal infertility factor, revealed intrauterine pregnancy rates of 43.6%, 34.0%, and 19.4% in mild, moderate, and severe disease, respectively [27]. In the most recent research by Nian et al., natural pregnancy rate within 2 years after neosalpingostomy for mild hydrosalpinx was 50%, 17.39% for moderate, and 15.6% for severe hydrosalpinx [58].

Unfortunately, reconstructive tubal surgery can not only result in desired intrauterine pregnancy, but also in ectopic pregnancy. The tubal pregnancy rate correlates with the severity of tubal damage achieving 2–8% in good-prognosis patients and up to 17% in women with poor prognosis [23,61]. According to Chu et al., the pooled ectopic pregnancy rate after neosalpingostomy for hydrosalpinx is 10% [60].

## 6. Conclusions

Restorative tubal surgery is still an acceptable and widely applied treatment option for tubal factor infertility despite the increase in usage of artificial reproductive technologies [2,5,63]. The estimated live-birth rate after distal tubal surgery varies from 9% for women with severe tubal damage to 69% in cases of mild disease [5]. Extrapolated data from the National Assisted Reproductive Technology Registry from 2020 reveal a 28.2% live-birth rate per cycle initiated in patients across all ages with tubal factor infertility [64]. However, comparing the outcomes of IVF and distal tubal surgery means not only looking at the numbers: interpretation is much more complex. Results of repair tubal surgery are mostly reported per patient as cumulative clinical pregnancy rate and/or live-birth rate over a set period of observation, e.g., 1 year, 2 years, or even more. In contrast, the ART success rates are published on a per-cycle basis, possibly with cumulative outcomes after several cycles of treatment, which may include fresh and frozen embryo transfers [2,6]. The lack of consistency in reporting success rates is one of the two main reasons why there are no adequate trials objectively comparing the reproductive outcomes after tubal surgery and ART. The second one is the questionable validity of classification systems used for the assessment severity of tubal damage [5].

It is important to emphasize that IVF does not restore tubal function and an infertile couple after the procedure remains infertile. Tubal surgery may offer a permanent cure in selected groups of patients, who can attempt pregnancy every cycle and conceive many times. Conception rarely happens immediately after surgical treatment. Pregnancy rate increases gradually in subsequent cycles, achieving a plateau at 24 months [23,60]. This is explained by the time needed for epithelial regeneration within tubal mucosa [23]. The majority of ART pregnancies, including cumulative cycles, happen within 1 year [6]. Regardless of this, published data show very high dropout rates after unsuccessful procedure: 74% after the first, 61% after the second, and 69% after the third attempt [65]. Decisions on treatment discontinuation are made because of disappointment and psychological stress [66].

Apart from a good per-cycle success rate, the main advantage of IVF is the avoidance of surgery [6]. Its disadvantages include costs, risk of ovarian hyperstimulation syndrome and multiple pregnancies, as well as a higher incidence of adverse perinatal outcomes such as: preterm delivery, intrauterine growth restriction, congenital malformations, and perinatal mortality [67,68,69,70,71,72,73,74,75]. The disadvantages of reconstructive tubal surgery are potential perioperative complications, e.g., damage of inner structures, postoperative pain, bleeding, infections, and adverse reactions to anesthesia. However, the overall risks of surgery are very small when using laparoscopic techniques and are compensated with the advantages of a minimally invasive approach. The risk of ectopic pregnancy after salpingoneostomy is higher compared to patients with tubal factor after ART: 2.0–17.4% vs. 2.1–11% [5,19,23,60,61].

There is no doubt that the treatment of tubal infertility has shifted towards IVF; however, many couples refuse it for psychological, ethical, religious, or financial reasons [76]. As a result, many gynecologists no longer perform corrective tubal procedures, although in well-selected cases, reproductive outcomes after surgery outperform those of ART. There is a growing risk that reproductive clinicians are becoming deskilled, and trainees do not have a chance to gain enough experience and develop the technical skills necessary for successful surgical intervention. Consequently, for patients with tubal factor infertility, the full range of treatment options are not always available, and the fundamental principle of individualized therapy is slowly being replaced by an “ART for everything” approach. To counteract this worrying trend and offer patients optimal tailored treatment meeting their individual goals and needs, high-quality trials comparing surgery vs. IVF and training of the next generation of reproductive surgeons are becoming imperative.

## Figures and Tables

**Table 1:** Winston and Margara staging system of tubal disease [23].

Stage I	Thin-walled hydrosalpinx with little or no fibrosis. Mucosa thrown into folds with no flattened areas. Adhesions (if present) flimsy and limited to the ampulla and ovary only. Ovary present and mainly free.
Stage II	Thick-walled hydrosalpinx with good mucosa. Mucosa flattened with attenuated or few folds but thin-walled areas. Mucosal fold markedly adherent in lumen. Fibrous thick adhesions involving tube and/or ovary. Ovary present and mainly free.
Stage III	Combination of thick-walled hydrosalpinx with marked mucosal damage or thick fibrous adhesions. Clean hydrosalpinx with thin wall but with nodularity of patent isthmus. Ovary incarcerated against pelvic side wall or absent on that side.
Stage IV	Tubo-ovarian mass or fibrous, adherent hydrosalpinx with incarcerated ovary and/or isthmic damage.

**Table 2:** Hull and Rutherford classification of tubal/pelvic disease [25].

Minor disease/Grade I
Tubal fibrosis absent even if occluded (proximally) Tubal distension absent even if occluded (distally) Mucosal appearances favourable Adhesions (peritubal–ovarian) flimsy
Intermediate disease/Grade II
Unilateral severe tubal damage With or without contralateral minor disease “Limited” dense adhesions of tubes and/or ovaries
Severe disease/Grade III
Bilateral severe tubal damage Tubal fibrosis extensive Tubal distension >1.5 cm Mucosal appearance abnormal Bipolar occlusion “Extensive” dense adhesions

## Data Availability

Not applicable.

## Abbreviations

ART (assisted reproductive technology), IVF (in vitro fertilization), PID (pelvic inflammatory disease), CT (computed tomography), US (ultrasonography), MR (magnetic resonance), U.S. FDA (The United States Food and Drug Administration).
